# Effect of Blending Ratio and Fermentation Time on the Quality and Acceptability of Injera Produced from a Composite of Teff, Maize, and Potato Flours

**DOI:** 10.1002/fsn3.71606

**Published:** 2026-03-08

**Authors:** Yengus Lake Cherinet, Mesfin Wogayehu Tenagasahw, Birhanu Ayitegeb Ambaw, Aynadis Molla Asemu, Wendu Hilemichael Hilemeskel, Behailu Bisenebit Mossie, Zenamarkos Bantie Sendekie

**Affiliations:** ^1^ Food Engineering, Faculty of Chemical and Food Engineering Bahir Dar Institute of Technology, Bahir Dar University Bahir Dar Ethiopia; ^2^ Bahir Dar Food and Nutrition Center Bahir Dar Institute of Technology, Bahir Dar University Bahir Dar Ethiopia

**Keywords:** blending ratio, fermentation time, injera, proximate composition, sensory acceptability

## Abstract

Injera, Ethiopia's traditional fermented flatbread, is made primarily from teff flour and water, characterized by its soft, spongy texture and slightly sour taste that develops during natural fermentation. This study aims to explore how blending ratios and fermentation times impact the quality of injera made with teff, maize, and potato flours. The research used a factorial design with two factors (blending ratio and fermentation time) arranged in a completely randomized treatment structure. The proximate compositions of all raw sample flours were significantly different at a 0.05 significance level. The results demonstrate that the addition of maize and potato flours in teff injera and subjecting the batter to extended fermentation (72 h) can effectively modify the nutritional profile of injera. Composite formulations, particularly BR1, offer potential advantages in terms of increased fiber content and reduced energy value, which may help address rising injera costs while maintaining acceptable nutritional quality. The study showed that blending teff with maize and potato flours, along with controlled fermentation times, significantly affects the physicochemical, nutritional, microbial, and sensory qualities of injera. BR1 fermented for 72 h is recommended, as it offers a shelf‐life and nutritionally beneficial alternative to injera made from teff.

## Graphical abstract

This study evaluates the effects of blending teff with maize and potato flours and varying fermentation time on injera quality. The optimal formulation (70% teff, 15% maize, 15% potato) with 72 h fermentation improved nutritional value, fiber content, and sensory and microbial quality, providing a cost‐effective alternative to 100% teff injera.

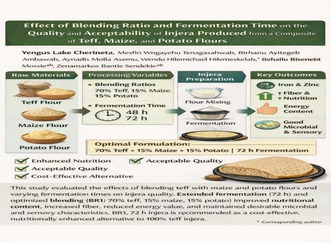

## 1 Introduction

Injera, a traditional Ethiopian fermented flatbread made primarily from teff flour and water, is characterized by its soft, spongy texture and slightly sour taste that develops during natural fermentation. Injera is a thin, round, spongy, pancake‐like flatbread that forms when CO_2_ is produced during fermentation and escapes during baking, holds cultural significance and is a staple in many households; it also has a tiny honeycomb‐like structure on the top surface (Anberbir et al. 2023). Nowadays, Injera is recognized worldwide for its gluten‐free qualities and is a fermented food that is extensively consumed in Ethiopia and Eritrea, but also by Ethiopian and Eritrean diaspora communities around the world (Asrat 2022).

Teff is a small, nutrient‐rich cereal grain from Ethiopia and Eritrea, essential for making injera. It thrives in various climates, including drought‐prone areas, and is packed with dietary fiber, protein, and a superbly balanced and full complement of amino acids, especially glutamic acid/glutamine (3.88 g/100 g), leucine/isoleucine (2.29 g/100 g), threonine (1.41 g/100 g), and is rich in essential minerals like calcium and iron, compared to other cereals. The exceptional nutritional composition of teff is the reason for its rising popularity and interest (Abebe et al. 2025). Maize is a widely grown cereal crop rich in carbohydrates and provides energy, along with dietary fiber, vitamins, and minerals, and is experiencing a steady increase in demand both domestically and internationally. However, Ethiopia's current teff production remains insufficient to meet this growing demand (Goersch et al. 2019). Potato is a starchy, versatile root tuber crop that is one of the world's most important food sources, rich in carbohydrates, mainly as starch, and also provides vitamin C, potassium, dietary fiber, and small amounts of protein, each bringing unique characteristics to the table (Degafe 2020).

In recent years, increasing demand for diverse and nutrient‐dense foods has driven greater interest in alternative and composite flour formulations. However, teff production has not kept pace with this demand due to several constraints, including the absence of high‐yielding improved varieties, fragmented traditional farming systems, substantial post‐harvest losses, and limited public investment in the sector (Goersch et al. 2019). These days, different cereals such as wheat, sorghum, millet, maize, rice, barley, amaranths, and quinoa are combined with teff to produce injera. Nevertheless, it is difficult to determine the injera sensory characteristics made from the composite flours (Fikadu et al. 2019). By blending the flours of these grains/tuber, aiming to create a product that not only addresses nutritional needs but also enhances the sensory experience of Injera. Additionally, fermentation, a critical step in the preparation of injera, plays a pivotal role in determining its texture, flavor, and overall quality (Anberbir et al. 2023).

This investigation seeks to contribute valuable insights into the optimal blending ratios and fermentation times that yield Injera with acceptable attributes, meeting both cultural expectations and modern dietary preferences. So, this proposal aims to delve into the intricate interplay of blending ratio and fermentation time on the quality attributes of Injera, specifically when prepared using a combination of teff, maize, and potato flours. This study aims to elucidate the collective influence of these variables on the quality of injera, addressing a significant gap in understanding how the incorporation of potato flour alters the nutritional composition, sensory attributes, and overall quality of injera, thus contributing to refining its production and nutritional enhancement.

## 2 Materials and Methods

### 2.1 Raw Material Collection

Teff, maize and potato samples with known variety Teff (Tseday), Maize (Keylaba) and Potato tuber (Abalo) were collected from Amhara seed enterprise. Maize grains of a recent harvest (year of harvest 2024) with uniform kernel characteristics were purchased from Bahir Dar market from a known grain retailer.

### 2.2 Sample Preparation

Teff flour was prepared by manual winnowing to remove light impurities, hand picking, and traditional destoning, as done in the households, and then milled using a mini mill (model OLISLAB3200). Maize flour was prepared after a thorough cleaning by sieving, winnowing, and hand picking of the maize grains to remove any dirt, debris, or other impurities, followed by grinding the grains using a mini laboratory mill (model OLISLAB3200). Potato tubers were washed, peeled, trimmed, sliced, and immediately blanched for 5 min in boiling water. Then, they were dried in a cabinet dryer at 60°C–65°C for 8–10 h and finally milled (model OLISLAB3200) to produce potato flour. All the flour types were then packed in a polyethylene bag to preserve the product until used for the experiment (Abebaw 2020).

### 2.3 Experimental Design

The experimental design was carried out using a two factorial design experiment conducted using a completely randomized design (CRD). The experiment consisted of two factors: blending ratio (BR) and fermentation time (FT). The BR of teff, maize, and potato flour had four levels (100:0:0, 70:10:20, 70:15:15, and 70:20:10), respectively, and fermentation time (FT) was conducted at three levels (60, 72, and 84 h) with triplicates. The proximate compositions, mineral contents, and functional property of flours and the composite bread were examined using this design paradigm.

### 2.4 Preparation of Fermented Dough and Baking of Injera

The injera was prepared using the method described by (Anberbir et al. 2023). The process involved starter preparation (“Ersho”), dough making, fermentation, and baking of the dough. The starter preparation was similar to sourdough starter preparation. Water and flour were combined in a 2:1 ratio and left to ferment undisturbed in a warm, dark place for up to 84 h before being used for making injera. The dough was made by mixing flour and water in a 1:2 ratio with about 160 mL of “Ersho” (starter culture). The dough was then kneaded by hand for 5 min and kept covered in a bowl at ambient conditions in the food processing laboratory.

Fermentation steps were carried out at 60, 72, and 84 h at room temperature (around 25°C). About 70 mL of supernatant was discarded, and the dough was thinned by adding 200 mL of clean water. The dough was left covered for 15 min to allow further fermentation. Abseit, which served as a binder, was prepared by boiling a mixture of 1 part thin dough to 3 parts water (about 120 g) on a hot oven and then cooling it to about 45°C (Yegrem et al. 2021). The cooled abseit was added to the thinned batter, mixed carefully, and left for 3 h for secondary fermentation until it rose and became ready for baking. The injera was baked by pouring about 500 mL of batter in a circular motion from the outer perimeter toward the center onto a hot, smooth, round baking griddle called a “metad” (at around 220°C) for 3 min.

### 2.5 Physicochemical Properties of Flour and Injera

The moisture content was determined using the oven‐drying method (PHG‐9140, China) according to AOAC (Ayalew et al. 2024) with the official method number 925.10. The crude protein content was determined by the Kjeldahl method (DK6, VELP Scientific, Italy) according to AOAC (Ayalew et al. 2024) with the official method number 920.87. The crude fat was determined by the Soxhlet extraction method (heater model‐98‐I‐BN, China) according to AOAC (Ayalew et al. 2024) using the official method number 920.39. In this method, hexane was used as the extraction solvent. The crude fiber content was determined by Dahmardeh (2012) using the official method number 962.09, with the acid and base digestion method. The total ash content was determined using a muffle furnace (FHX‐05, Korea) according to AOAC (Ayalew et al. 2024) with the official method number 923.03. The total percentage of carbohydrates (CHO) was determined by using the difference method according to (Chukwuma et al. 2018).

(1) $$\text{Total}\;\mathrm{CHO}\;\left(\%\right)=100-\left(\%\mathrm{MC}+\%\mathrm{CFC}+\%\mathrm{PC}+\%\mathrm{AC}+\%\mathrm{FC}\right)$$

The total energy content of the injera samples was determined according to AOAC, (2000) by multiplying the mean value of crude fat (*F*), total carbohydrate (CHO), and crude protein (P) by the Atwater factor 9, 4, and 4 respectively.

(2) $$\text{Energy}\;\left(\text{Kcal}\;100\;g\right)=\left(P\times 4\right)+\left(F\times 9\right)+\left(\mathrm{CHO}\times 4\right)$$

The pH of the fermented dough was determined using the method of using (Fangye Zeng 2023), directly by dipping the pH meter into homogenized mixture slurries before fermentation and after the end of each fermentation period. The total titratable acidity of dough and injera was determined according to the methods described (Berhe 2014)
(3)

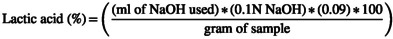




### 2.6 Determination of Mineral Content

The iron, calcium, and zinc content were determined using the official method of Flame Atomic Absorption Spectrophotometry, 923.03. A 2 g sample was digested using a Kjeldahl digester with 10 mL of concentrated HNO3 and 4 mL of 70% HClO4. The resulting solution was quantitatively transferred into a 100 mL volumetric flask. A calibration curve was prepared by plotting the absorption or emission values against the metal concentration in ppm. The solution was then sprayed into an atomic Absorption Spectrophotometer at 248.3 nm to determine the concentration of iron, calcium, and zinc. The readings were taken from the graph, which depicted the metal concentrations corresponding to the absorption or emission values of the samples and the blank.

(4) $$\text{Metal content}=\left(\mathrm{mg}/100g\right)=\left(\left(A-B\right)\times V\right)/10W)\times 100)$$

where—W = Weight of sample in (gm), V = Volume of extract (mL), A = Concentration of Sample solution (μg/mL), B = Concentration of blank solution (μg/mL).

### 2.7 Determination of Functional Properties of Flour

#### 2.7.1 Bulk Density (BD)

Bulk density (BD) was determined according to the methods described by (Anberbir et al. 2023). The flour sample was transferred to a pre‐weighed measuring cylinder, and the new weight was recorded. The volume occupied by the flour in the measuring cylinder was also recorded.

(5) $$\text{Bulk density}\;\left(\frac{g}{\mathrm{mL}}\right)=\frac{\text{Mass of Sample}}{\text{Volume of Cylinder}}$$

#### 2.7.2 Water Absorption Index (WAI)

The water absorption index (WAI) was determined according to the methods described (Berhe 2014). The sample 2.5 g was suspended in 30 mL of distilled water in a tared 50 mL centrifuge tube and shaken for 30 min. The sample was then centrifuged for 10 min at 3000 rpm. The clear supernatant of the centrifugation was transferred into a pre‐dried glass beaker and weighed for the estimation of the water solubility index. The gel remaining in the centrifuge tube was weighed.

(6) $$\text{Water absorption index}\;\left(\%\right)=\frac{\text{Weight of sediments}\;}{\text{Weight of}\;\mathrm{dry}\;\text{sample}}100$$

#### 2.7.3 Water Solubility Index (WSI)

The water solubility index (WSI) was determined by taking the supernatant preserved from the WAI measurement according to (Nargis Yousf 2017), which was evaporated at 96°C overnight.

(7) $$\mathrm{WSI}\;\left(\%\right)=\frac{\text{Weight of dissilve solids in the supernatant}}{\text{Weight of}\;\mathrm{dry}\;\text{sample}}\times 100$$

### 2.8 Microbial Quality Analysis of Injera

The total aerobic plate count and total yeast‐mold count were carried out on injera samples during (fresh), 24, 72, and 120 h of ambient temperature storage using the procedure of (Nibret 2024).

### 2.9 Sensory Evaluation

The sensory acceptability of the injera samples was determined according to the methods described by (Anberbir et al. 2023). A total of 50 semi‐trained panelists were randomly selected from Bahir Dar Institute of Technology (BiT) staff. All samples were subjected to sensory evaluation after 2 h of baking. The evaluation was carried out based on sensory attributes: color, texture, taste, eye uniformity, top and bottom surface (degree of being powdery and sticky), rollability, and overall acceptability using a seven‐point hedonic scale, where 1 = dislike very much, 2 = dislike moderately, 3 = dislike slightly, 4 = neither like nor dislike, 5 = like slightly, 6 = like moderately, and 7 = like very much. The injera sample was served on a white plate, arranged, and coded randomly. During the evaluation, panelists were instructed to rinse their mouths with water between each sample tasting. The study was reviewed and approved by the Bahir Dar Institute of Technology, Bahir Dar University, Research Ethics Review Board (IRB) under approval number BiT‐IRERB/067/2026, and informed consent was obtained from each subject prior to their participation in the study.

### 2.10 Data Analysis

The statistical analysis of the data from each experiment was conducted using Minitab software, and the results were expressed as mean ± standard deviation (SD). A two‐way analysis of variance (ANOVA) was used to investigate both the independent and combined effects of each factor on response variables. Further, mean comparisons were conducted by significant difference (Tukey's test), and statistical significance was set at a 95% confidence level (*p* < 0.05). Sigma Plot software was used to create all graphs.

## 3 Results and Discussion

### 3.1 Proximate Composition

The proximate compositions of maize, potato, teff, and their composite flours are presented in Table 1. Notable variations were observed in the nutritional profiles of maize, potato, and teff flours, which may influence their suitability for specific dietary applications and the development of fermented foods. The moisture content of maize, potato, and teff flours was significantly affected by the type of flours (*p* < 0.05). Among the flours, potato exhibited the highest moisture content (8.36%), followed by teff (7.93%) and maize (5.37%). The elevated moisture content in potato flour may be attributed to its high starch content, which binds more water during processing and storage (Deng et al. 2024). Starch granules, especially in potatoes, have a strong affinity for water due to their high amylopectin content, increasing moisture retention (Bikila 2020). Similarly, the moisture content of the composite flour was significantly affected by the blending ratio (*p* < 0.05). The control (BRC) retained the highest moisture content (7.93%). Teff holds more water than maize‐based blends due to its higher protein and fiber content, hydrophilic groups, and smaller starch granules. Maize flour's higher starch and lower protein and fiber levels reduce moisture retention, resulting in greater water (Sumbo H 2014). It was observed that the moisture content of the composite flours decreased as the amount of potato flour was reduced and the proportion of maize flour was increased in the composite flours. This behavior is likely because of the higher maize content, which has a lower water retention capacity (Twinomuhwezi et al. 2020), combined with the greater moisture retention capacity of potato flour (Deng et al. 2024).

**TABLE 1:** Proximate composition of raw materials and composite flours.

Treatments	MC (%)	Crude protein (%)	Fiber (%)	Fat (%)	Ash (%)	Carbohydrate (%)
Teff	7.93 ± 0.7^a^	7.8 ± 0.1^a^	2.03 ± 0.02^a^	2.86 ± 0.04^b^	3.02 ± 0.87^a^	76.43 ± 0.76^b^
Maize	5.4 ± 0.3^b^	6.4 ± 0.1^b^	1.55 ± 0.08^b^	4.24 ± 0.03^a^	3.00 ± 0.02^a^	79.42 ± 0.18^a^
Potato	8.4 ± 0.1^a^	8.1 ± 0.1^a^	1.08 ± 0.007^c^	0.16 ± 0.03^c^	2.95 ± 0.01^b^	79.35 ± 0.16^a^
CV	1.59	1.41	0.69	0.66	0.31	0.20
*p* value	0.000	0.000	0.000	0.000	0.007	0.000
BRC	7.93 ± 0.73^a^	3.02 ± 0.87^a^	2.03 ± 0.02^a^	2.86 ± 0.04^a^	7.78 ± 0.03^a^	76.42 ± 0.04^d^
BR1	7.14 ± 1.41^ab^	2.32 ± 0.03^b^	1.81 ± 0.01^c^	2.47 ± 0.02^b^	7.73 ± 0.01^a^	78.52 ± 0.01^c^
BR2	6.29 ± 0.62^b^	2.50 ± 0.46^ab^	1.82 ± 0.02^c^	2.67 ± 0.03^b^	7.62 ± 0.05^b^	79.13 ± 0.06^b^
BR3	5.51 ± 0.44^b^	2.25 ± 0.29^b^	1.87 ± 0.01^b^	2.89 ± 0.03^a^	7.51 ± 0.06^c^	79.99 ± 0.01^a^
CV	6.48	1.21	0.57	0.62	0.15	0.01
*p* value	0.010	0.001	0.000	0.000	0.00	0.00

*Note:* Mean ± standard deviation; Means with Different superscripts within a column differ significantly (*p* < 0.05). Where: BR, blending ratio; BR1, (70% Teff: 10% maize: 20% potato), BR2, (70% Teff: 15% maize: 15% potato), BR3, (70% Teff: 20% maize: 10% potato), BRC, (100% Teff:0% maize: 0% potato).

Protein content is a key determinant of the nutritional quality of flours. Potato had the highest protein content (8.10%), slightly surpassing teff (7.82%), while maize had the lowest (6.42%). The relatively high protein content in potato is primarily due to its rich amino acid profile, particularly lysine, which is often limited in cereals (Buzera et al. 2024). Despite this, teff remains a nutritionally superior grain owing to its well‐balanced amino acid composition and gluten‐free nature (Hu et al. 2023). Thus, the higher protein content in potato is linked to its biological role as a nutrient‐rich storage organ and its diverse amino acid composition, which together contribute to a greater and more nutritionally complete protein level compared to teff and maize. Teff and maize are cereal grains with starch‐based endosperms and deficient zeins, reducing protein quality and quantity. Teff has a balanced amino acid profile but slightly lower protein content than potato (Satheesh and Fanta 2018).

The crude fat contents of teff, maize, and potato flours were 2.86%, 4.24%, and 0.16%, respectively (Table 1). Accordingly, teff provides a moderate fat level that is perfect for fermentation, but maize flour features the largest fat concentration, which adds to flavor and calorie density. In contrast, potato flour contains very little fat, and its primary role in injera production is to enhance flexibility and improve moisture retention (Mohammed 2025).

These observations are supported by (Baye 2014) and Dabi et al. (2024) who pointed out that teff's moderate fat content makes it ideal for fermentation and that it helps create spongy and elastic injera by retaining more gas during the fermentation process. According to Baye (2014), maize's increased fat content enhances flavor and energy density, but excessive use may prevent fermentation. Buzera et al. (2023) indicate that potato flour's low‐fat content is consistent with its main function as a starch source for flexibility and moisture retention. Combining these flours can improve the quality of injera by striking a balance between flavor, texture, and cost effectiveness.

Dietary fiber is an essential nutritional component that enhances digestion and metabolic health. Among the ingredients, teff had the highest fiber content (2.03%), followed by maize (1.55%) and potato (1.08%) (Table 1). The high fiber content in teff is due to its whole‐grain nature, as it retains more bran and germ components (Satheesh and Fanta 2018). In contrast, potato has less fiber, as much of its starch is in the form of digestible polysaccharides, and maize contributes moderate fiber levels. In the composite flours, BR1 (1.81%) and BR2 (1.82%) maintained high fiber content, reflecting the influence of teff's fiber, while BR3 (1.87%) had slightly higher fiber content due to a higher percentage of potato in the blend. The significant differences (*p* < 0.05) in fiber content indicate that higher maize and potato ratios reduce the total fiber content of the blends (Satheesh and Fanta 2018).

Ash content reflects the total mineral composition of the flour. Teff had the highest ash content (3.02%), followed by maize (3.00%) and potato (2.95%). The high ash content in teff is linked to its rich mineral composition, particularly iron, calcium, and zinc (Tafese Awulachew 2020).

In composite flours, BRC (3.02%) retained the highest ash content, similar to pure teff, indicating its superior mineral composition. The ash content of BR1 (2.32%), BR2 (2.50%), and BR3 (2.25%) decreased as the proportion of maize and potato increased, reflecting the lower mineral content of these ingredients. Blending ratio had significant effects (*p* < 0.05) on the ash content of composite flours. Carbohydrates are the primary energy source in flour‐based products. Maize (79.42%) and potato (79.35%) had the highest carbohydrate contents, while teff had a slightly lower content (76.43%). This variation is expected as maize and potato are predominantly starch‐rich ingredients, while teff contains more fiber and protein, slightly reducing the overall carbohydrate percentage (Awulachew 2020).

### 3.2 Functional Properties of Flours

The physical properties of teff, maize, and potato flours play a crucial role in injera making, and significant differences were observed in bulk density (BD), water absorption index (WAI), and water solubility index (WSI) (Table 2). Potato flour, with its highest BD (0.58 g/mL) compared to teff (0.50 g/mL) and maize (0.33 g/mL), may offer advantages in injera making related to batter consistency and gas retention during fermentation. The higher BD contributes to batter stability, which is desirable for injera fermentation and cooking. Furthermore, potato flour's highest WAI (317.86%) and WSI (14.74%) (*p* < 0.05) suggest increased water retention and soluble starch availability, potentially resulting in a softer, more flexible injera texture. Maize flour's high WAI (313.99%) might contribute to a similar texture if incorporated into injera blends (Table 2). In contrast, teff flour, exhibiting the lowest WAI (209.42%) and WSI (7.11%, *p* < 0.05), traditionally provides the characteristic slightly tangy flavor and slightly firm, porous structure of injera. While teff alone yields the authentic injera, blending it with maize or potato flour could alter the final product's texture, flexibility, and overall sensory attributes, requiring careful consideration of the blend ratios to maintain desirable injera qualities (Ashenafi 2016).

**TABLE 2:** Functional properties of raw materials and composite flours.

Treatments	BD (gm/mL)	WAI (%)	WSI (%)
Teff	0.50 ± 0.01^a^	209.42 ± 4.04^b^	7.11 ± 0.43^c^
Maize	0.33 ± 0.12^b^	313.99 ± 13.49^a^	8.65 ± 0.31^b^
Potato	0.58 ± 0.02^a^	317.86 ± 15.85^a^	14.74 ± 0.25^a^
CV	1.13	1.93	1.7
*p*‐value	0.011	0.000	0.000
BR1	0.49 ± 0.002^a^	258.07 ± 0.59^a^	8.78 ± 0.02^a^
BR2	0.48 ± 0.005^ab^	250.49 ± 0.52^b^	8.74 ± 0.02^ab^
BR3	0.47 ± 0.002^b^	244.53 ± 0.85^c^	8.69 ± 0.02^b^
BRC	0.5 ± 0.01^a^	208.95 ± 0.57^d^	8.14 ± 0.03^c^
CV	0.32	0.21	0.17
*p*‐value	0.00	0.00	0.00

*Note:* Mean ± standard deviation; Means with different superscripts within a column differ significantly (*p* < 0.05). where: BR = blending ratio; BR1 = (70% Teff: 10% maize: 20% potato), BR2 = (70% Teff: 15% maize: 15% potato), BR3 = (70% Teff: 20% maize: 10% potato), BRC = (100% Teff:0% maize: 0% potato).

Depending on the desired outcome (increased softness, improved rollability), incorporating these alternative flours could offer opportunities to modify and diversify the injera‐making process. BR3 has the lowest bulk density, indicating it is lighter and fluffier, ideal for foods requiring lower weight (weaning or snack foods). BRC and BR1 have the highest bulk density, indicating more compact flour suitable for energy‐dense or solid food applications. BR1 has the highest WAI, indicating better hydration and swelling properties valuable for bakery, porridges, and soups. BRC shows significantly lower WAI, likely due to higher Teff content (Teff has lower WAI compared to potato and maize). A gradual decline in WAI is observed from BR1 to BRC, showing the impact of ingredient composition on water‐holding capacity.

BR1 has the highest WSI, suggesting more soluble components (starch degradation, simple sugars, soluble fiber). BRC has the lowest WSI, indicating fewer soluble, likely because of teff's high insoluble fiber content. High WSI is favorable for digestibility and may influence mouthfeel in instant or rehydrated foods (Yang 2024).

### 3.3 Mineral Content Analysis of Flours

The mineral composition analysis reveals significant differences in calcium (Ca), iron (Fe), and zinc (Zn) concentrations across teff, maize, and potato flours, which directly impacts their suitability for injera production (Table 3). Teff flour (BRC) exhibits markedly elevated levels of Ca (3.75) and Fe (10.0 mg/100 g), establishing it as a superior source of these essential micronutrients in injera (Leykun et al. 2020). Conversely, maize flour presents comparatively lower Ca (2.4) and Fe (2.38 mg/100 g) concentrations, than teff and potato potentially requiring supplementation when used in injera formulations. Potato flour, while possessing intermediate Ca (2.55) and Fe (3.86 mg/100 g) levels, displays notably higher Zn content (2.94 mg/100 g) (Gloria and Abbas 2018), offering a potential pathway for zinc enrichment in injera.

**TABLE 3:** Mineral compositions of raw materials and composite flours.

Individual	Ca (mg/100 g)	Fe (mg/100 g)	Zn (mg/100 g)
Teff	3.75 ± 0.07^a^	10.03 ± 0.19^a^	2.21 ± 0.05^b^
Maize	2.4 ± 0.03^c^	2.38 ± 0.04^c^	1.41 ± 0.05^c^
Potato	2.55 ± 0.07^b^	3.86 ± 0.09^b^	2.94 ± 0.10^a^
CV	1.27	1.48	2.07
*p* value	0.000	0.000	0.000
BR1	2.83 ± 0.04^c^	6.6 ± 0.12^c^	1.78 ± 0.04^b^
BR2	3.22 ± 0.04^b^	6.73 ± 0.09^c^	1.94 ± 0.04^b^
BR3	2.96 ± 0.08^c^	7.11 ± 0.03^b^	1.9 ± 0.10^b^
BRC	3.75 ± 0.07^a^	10.03 ± 0.19^a^	2.21 ± 0.05^a^
CV	1.12	0.35	2.07
*p* value	0.000	0.000	0.000

*Note:* Mean ± standard deviation; Means with different superscripts within a column differ significantly (*p* < 0.05). where: BR = blending ratio; BR1 = (70% Teff: 10% maize: 20% potato), BR2 = (70% Teff: 15% maize: 15% potato), BR3 = (70% Teff: 20% maize: 10% potato), BRC = (100% Teff:0% maize: 0% potato).

The formulation of composite flours through the blending of these ingredients results in a predictable modulation of the mineral profile, influencing the nutritional quality of injera. The incorporation of teff flour elevates Ca and Fe concentrations in injera, while the inclusion of potato flour enhances Zn content (Table 3). These findings indicate that the strategic manipulation of flour blending ratios presents a viable approach for tailoring the mineral composition of injera to meet specific nutritional objectives (Leykun et al. 2020).

These results are consistent with established knowledge regarding the mineral composition of these staple food sources (Nyachoti et al. 2021) and their implications for dietary intake. The recognized iron and calcium richness of teff (Leykun et al. 2020), the need for mineral fortification in maize‐based diets often used in injera blends, and the potential of potato flour as a zinc‐enhancing ingredient in injera are all corroborated by the present analysis.

### 3.4 Physicochemical Analysis of Injera

Effect of blending and fermentation time on pH and TA of Dough were presented in (Table 4), which demonstrates that the pH of dough significantly decreases as fermentation time increases, regardless of the blending ratio of teff, maize, and potato. Initially, at *t* = 0, all dough samples have relatively high pH values, with the control sample (BRC: 100% teff: 0% maize: 0% potato) showing the highest pH, followed by the blends BR1 (70:10:20), BR2 (70:15:15), and BR3 (70:20:10). These differences at the start are statistically significant, as indicated by the different letters above the bars, and are likely due to the higher buffering capacity and unique biochemical composition of teff compared to maize and potato. As fermentation progresses to 60, 72, and 84 h, a sharp decline in pH is observed across all samples, with values converging between 3.5 and 4.0. This drop is attributed to the metabolic activity of lactic acid bacteria, which convert available carbohydrates into organic acids, primarily lactic acid, thereby lowering the pH of the dough (Bharadwaj 2020).

**TABLE 4:** Effect of blending ratio and fermentation time on pH, dough force, and titratable acidity of dough.

	BR	BRC	BR1	BR2	BR3	CV	*p*
pH	0 h	6.50 ± 0.15ᵃ	6.30 ± 0.14ᵃᵇ	6.20 ± 0.13ᵇ	6.10 ± 0.14ᵇ	2.27	< 0.001
	60 h	3.90 ± 0.10ᶜᵈ	3.80 ± 0.09ᶜᵈ	3.70 ± 0.08ᶠᵍ	3.60 ± 0.08ᵇᵉᶠ	2.35	< 0.001
	72 h	3.60 ± 0.08ᵍ	3.50 ± 0.07ᵍ	3.60 ± 0.07ᶠᵍ	3.50 ± 0.07ᵉᶠᵍ	0.02	< 0.001
	84 h	3.80 ± 0.09ᶜᵈᵉ	3.70 ± 0.09ᵈᵉᶠᵍ	3.60 ± 0.08ᶠᵍ	3.70 ± 0.09ᵉᶠᵍ	2.41	< 0.001
Dough force (N)	0 h	—	—	—	—		
	60 h	8.80 ± 0.30ᵇ	9.10 ± 0.35ᵇ	8.00 ± 0.32ᵇᶜ	11.00 ± 0.40ᵃ	3.67	< 0.001
	72 h	6.60 ± 0.25ᵈ	7.20 ± 0.30ᶜᵈ	6.40 ± 0.24ᶜᵈ	6.70 ± 0.25ᵈ	4.00	< 0.001
	84 h	6.70 ± 0.30ᵈ	7.00 ± 0.28ᶜᵈ	6.80 ± 0.26ᶜᵈ	7.80 ± 0.30ᵇᶜ	4.10	< 0.001
Titratable acidity (%)	0 h	0.50 ± 0.03ᵉ	0.48 ± 0.03ᵉ	0.50 ± 0.03ᵉ	0.52 ± 0.03ᵉ	6.03	< 0.001
	60 h	1.18 ± 0.05ᵇᶜ	1.10 ± 0.05ᶜᵈ	1.25 ± 0.05ᵃᵇ	1.20 ± 0.05ᵇᶜ	4.35	< 0.001
	72 h	1.24 ± 0.05ᵇᶜ	1.30 ± 0.06ᵃ	1.28 ± 0.05ᵃ	1.25 ± 0.05ᵇᶜ	4.19	< 0.001
	84 h	1.12 ± 0.04ᶜᵈ	1.02 ± 0.04ᵈ	1.20 ± 0.05ᵇᶜ	1.18 ± 0.04ᶜ	3.78	< 0.001

*Note:* Mean ± standard deviation; Means with different superscripts within a column differ significantly (*p* < 0.05). where: BR = blending ratio; BR1 = (70% Teff: 10% maize: 20% potato), BR2 = (70% Teff: 15% maize: 15% potato), BR3 = (70% Teff: 20% maize: 10% potato), BRC = (100% Teff:0% maize: 0% potato).

The convergence of pH values after extended fermentation suggests that, although the initial blend composition influences the starting pH, the fermentation process ultimately dominates the acidification outcome, resulting in similar final acidity levels regardless of the blend. This finding aligns with previous studies, which report that pure teff dough tends to be more acidic than composite blends at the initial stages; however, all formulations reach a similar acidic endpoint after sufficient fermentation. (Meskel et al. 2017). The reduction in pH is most rapid between 0 and 60 h, after which the rate of decrease slows, indicating that the bulk of acid production occurs early in fermentation, with lactic acid bacteria activity tapering off as the environment becomes more acidic and less favorable for their growth (Casado et al. 2017). Such results are important for optimizing fermentation protocols in injera and similar products, as they show that a range of teff, maize, and potato ratios can be used without significantly affecting the final product's acidity, provided fermentation is adequate. This acidification is also essential for flavor, shelf life, and microbial safety of the final product (Ashenafi 2006).

The graph shows that the titratable acidity (TA) of the dough increases significantly from 60 to 72 h of fermentation across all samples, with the highest TA values observed at 72 h. This increase is attributed to the active metabolism of fermentative microorganisms, particularly lactic acid bacteria, which convert available carbohydrates into organic acids such as lactic and acetic acids during fermentation. The accumulation of these organic acids is responsible for the observed rise in TA, a trend that is well documented in studies on cereal fermentation (Alemayehu et al. 2024).

After 72 h, there is a slight decrease or plateau in TA at 84 h. This pattern suggests that acidification reaches a peak as the microbial population either exhausts available fermentable substrates or is inhibited by the increasingly acidic environment, which limits further acid production (Yigzaw et al. 2021). The statistical groupings indicated by the letters above the bars confirm that these differences are significant, particularly between the 60 and 72 h time points, and less pronounced between 72 and 84 h. This trend is consistent with the typical fermentation dynamics observed in cereal‐based doughs, where the most rapid acidification occurs during the initial and mid‐stages of fermentation, followed by stabilization as the system approaches equilibrium (Yigzaw et al. 2022). The increase in TA is crucial for the development of the characteristic sour flavor, improved shelf life, and microbial safety of fermented products (Abebe et al. 2023).

The graph presents the titratable acidity (TA) of dough samples at initial time (before fermentation time) and three fermentation times: 60, 72, and 84 h. The data show a clear trend where TA increases significantly from 60 to 72 h across all samples, reaching its peak at 72 h, particularly in the BR1 group. This increase is attributed to the active fermentation process, during which lactic acid bacteria and yeasts metabolize available carbohydrates and produce organic acids, mainly lactic and acetic acids. This process of acid accumulation is well established in the literature, as it is a key factor in the development of flavor and preservation in fermented cereal products (Alemu et al. 2024). After 72 h, the TA either slightly decreases or stabilizes at 84 h, which suggests that the acidification process reaches a plateau. This phenomenon occurs because the fermentable substrates become depleted, and the increasingly acidic environment inhibits further microbial activity and acid production. Similar observations have been reported in studies on the fermentation of injera and other cereal‐based foods, where acidification slows or stops as the system approaches equilibrium (Kitaw et al. 2024).

The increase in TA during fermentation is crucial for the development of the characteristic sour taste, extended shelf life, and improved microbial safety of fermented products. These findings highlight the importance of optimizing fermentation time to achieve desired sensory and safety qualities in traditional fermented foods. The effect of blending ratio and fermentation time on the texture of injera is presented in (Table 4). The pure teff control (BRC 100:0:0) exhibited significantly higher firmness at T1 (60 h of fermentation), denoted by the statistical significance letter “a,” with a measured value of approximately 11 N (Table 4). This result confirms that traditional teff injera possesses greater firmness compared to composite flour blends. Firmness decreased with increasing fermentation time, likely due to the progressive breakdown of the protein matrix that contributes to dough structure and elasticity (Ashenafi 2006). Among the composite blends, BR2 (70:15:15) shows the highest firmness at T1, suggesting an optimal balance between maize and potato components.

The equal ratio appears to maintain better textural properties than when either ingredient dominates (Asrat 2021). BR3 (70:20:10), with the highest maize content, shows reduced firmness, supporting findings that “texture score decreased when the proportion of maize is increased (Asrat 2021). This occurs because maize has a larger starch granule size (20 μm) compared to teff's smaller granules (2‐6 μm), affecting dough rheology (Asrat 2021). For potato content, BR1 (70:10:20) shows moderate firmness but research indicates higher potato concentrations can improve overall acceptability despite slightly lower firmness (Yassin and Getu 2019).

The fermentation time of 60 h (FT1) consistently produces the firmest texture across all blends, while extended fermentation (T2 = 72 h, T3 = 84 h) generally reduces firmness as the protein structure weakens and gas production changes (Bikila et al. 2024). Despite BRC showing the highest firmness, blends with moderate potato and maize content fermented for 72–84 h often have better overall sensory acceptability, suggesting optimal injera requires balancing firmness with other desirable qualities (Yassin and Getu 2019).

The effects of blending ratio and fermentation time on the proximate composition of injera are presented in Table 5. Moisture content peaked at 71.12% in BR1 at 72 h of fermentation, which was significantly higher than that of the control sample (59.72% for BRC at 60 h; *p* < 0.05) (Table 5). The combined effects of fermentation time and potato flour blending synergistically enhanced water retention. This increase can be attributed to the high amylopectin and soluble fiber content of potato, particularly arabinogalactan and pectin, which form a hydrophilic matrix capable of retaining water during fermentation. Microbial activity (Lactobacillus species) hydrolyzes starch into dextrins, increasing soluble solids that further bind water (Włodarczyk 2021).

**TABLE 5:** Effect of blending ratio and fermentation time on proximate composition of injera.

BR	*t* (h)	MC (wb)	Ash (%)	Fiber (%)	Fat (%)	Protein (%)	CHO (%)
BR1	60	61.65 ± 1.09^ef^	3.41 ± 0.67^ab^	2.501 ± 0.275^cdef^	2.45 ± 0.02^d^	7.55 ± 0.03^c^	22.43 ± 1.91^b^
BR1	72	71.12 ± 1.60^a^	2.74 ± 1.14^ab^	3.152 ± 0.543^abc^	2.44 ± 0.02^d^	7.51 ± 0.01^def^	13.04 ± 2.6^g^
BR1	84	68.07 ± 0.37^b^	2.90 ± 0.29^ab^	2.48 ± 0.08^cdef^	2.43 ± 0.015^d^	7.48 ± 0.02^fgh^	16.64 ± 2.1^f^
BR2	60	63.62 ± 0.24^de^	4.18 ± 0.99^a^	3.63 ± 0.15^a^	2.66 ± 0.345^bc^	7.54 ± 0.01^cd^	18.37 ± 1.1^df^
BR2	72	65.69 ± 0.22^cd^	2.78 ± 0.02^ab^	3.20 ± 0.08^ab^	2.64 ± 0.03^c^	7.50 ± 0.01^defg^	18.18 ± 2.91^df^
BR2	84	66.65 ± 0.98^bc^	3.68 ± 0.47^ab^	2.368 ± 0.37^def^	2.63 ± 0.034^c^	7.47 ± 0.02^gh^	17.20 ± 2.71^ef^
BR3	60	62.49 ± 0.82^e^	3.68 ± 0.23^ab^	2.95 ± 0.14^abcd^	2.87 ± 0.035^a^	7 s.53 ± 0.01^cdf^	20.48 ± 2.32^c^
BR3	72	66.44 ± 0.80^bc^	1.91 ± 0.46^b^	2.21 ± 0.02^ef^	2.86 ± 0.03^a^	7.49 ± 0.01^efgh^	19.09 ± 2.0^d^
BR3	84	66.27 ± 0.12^bc^	3.88 ± 0.06^a^	1.85 ± 0.06^f^	2.84 ± 0.03^a^	7.45 ± 0.01^h^	17.71 ± 1.5^ef^
BRC	60	59.72 ± 0.66^f^	3.13 ± 0.11^ab^	2.66 ± 0.26^bcde^	2.84 ± 0.04^a^	7.79 ± 0.01^a^	23.86 ± 2.03^a^
BRC	72	65.36 ± 0.11^cd^	4.30 ± 1.03^a^	2.87 ± 0.07^bcde^	2.80 ± 0.01^ab^	7.77 ± 0.01^ab^	16.90 ± 0.91^f^
BRC	84	62.22 ± 0.04^e^	2.95 ± 0.54^ab^	3.15 ± 0.17^abc^	2.82 ± 0.04^a^	7.73 ± 0.01^ab^	21.13 ± 1.6^c^
CV		0.33	6.19	0.7	0.51	0.13	1.13
P‐Value	T	0.027	0.049	0.006	0.000	0.000	0.000

*Note:* Mean ± standard deviation; Means with Different superscripts within a column differ significantly (*p* < 0.05). Where: BR = blending ratio; BR1 = (70% Teff: 10% maize: 20% potato), BR2 = (70% Teff: 15% maize: 15% potato), BR3 = (70% Teff: 20% maize: 10% potato), BRC = (100% Teff: 0% maize: 0% potato).

Pressed potato fibers have been shown to exhibit a high water‐holding capacity (WHC), primarily due to a network of insoluble, noncellulosic cell wall polysaccharides, including pectic substances and arabinogalactan. Ramasamy et al. (2015) confirmed that the WHC of pressed potato fiber can reach up to 7.4 mL/g dry matter, a property that translates into increased moisture retention in composite foods such as injera (Ramasamy 2014). At 84 h, a decline in moisture content to 68.07% in BR1 is observed, likely due to over‐fermentation, which can degrade the pectin network and lead to syneresis (water expulsion). This trend is consistent with findings that enzymatic or microbial degradation of pectic polysaccharides reduces the WHC of potato fiber matrices (Ramasamy 2014).

The ash content, reflecting mineral composition, exhibited statistically significant variation among treatments (*p* < 0.05), influenced by fermentation duration. BR2 demonstrated the highest ash content (4.18%) at 60 h, while BR3 showed the lowest (1.91%) at 72 h (Table 5). The elevated ash in BR2 likely stems from maize's inherent mineral contribution, whereas the reduction in BR3 aligns with prolonged fermentation facilitating mineral leaching into the liquid phase and microbial utilization (Harcourt and State 2024). These dynamics are consistent with studies showing that fermentation can either concentrate solids or deplete soluble minerals, depending on microbial activity and substrate composition. Previous research has observed similar fluctuations, attributing ash variability to microbially mediated mineral solubilization and substrate‐specific responses. Compared to raw flours (teff: 2.8%; maize: 1.5%; potato: 1.0%), the slight increase in ash content in fermented injera suggests a net retention of mineral‐rich solids despite partial leaching of soluble minerals (Harcourt and State 2024).

The interaction of blending ratio and fermentation time had significant effect on the fiber content of injera (*p* < 0.05) Table 5, BR2 at 60 h showing the highest fiber content (3.63%) and BR3 at 84 h had the lowest fiber content (1.85%). The higher fiber in BR2 can be associated with the maize content, which is naturally higher in fiber compared to potato, while the observed decrease over time could be due to microbial enzymes degrading complex fiber structures during fermentation. This observation matches with the findings of (Dabi et al. 2024), who demonstrated that prolonged fermentation reduces fiber in cereal and tuber‐based fermented foods due to enzymatic hydrolysis. Considering that teff and maize are fiber‐rich while potato is lower, the observed fiber levels align with the expected blending effects (Anberbir et al. 2023).

The fat content showed significant differences among blending ratios but minimal changes across fermentation times: Table 5. BR3 maintained the highest fat content consistently (around 2.84%–2.87%), while BR1 remained lower (2.42%–2.45%). This trend is mainly explained by the higher fat content of maize flour compared to teff and potato. The minimal changes observed in lipid content across fermentation periods indicate that microbial lipolysis was not a predominant process, aligning with previous studies reporting that short‐term fermentation exerts limited effects on the lipid composition of cereal‐based foods (Anberbir et al. 2023).

The protein content of the product remained relatively high and stable, ranging from 7.45% to 7.79%, which is nutritionally favorable. The control group consistently exhibited the highest protein content, suggesting that the traditional formulation may better preserve or enhance protein concentration compared to composite blends than the BR1–BR3 treatments over time. The slight decline in protein for BR1–BR3 with increasing fermentation or processing time might be due to protein degradation, microbial consumption, or dilution effects. Yegrem et al. (2021) reported that the protein content of injera ranged from 11.78% to 18.84%, which is greater than the present findings. This may be due to the carbohydrate content of injera decreased significantly with longer fermentation times and higher potato proportions in the blend. In this study, the control (BRC) exhibited the highest carbohydrate values at 60 h (23.86%) and 84 h (21.13%), while the lowest was observed in BR1 at 72 h (13.04%) Table 5. Blends with higher maize content (BR3) tended to retain more carbohydrates than those with higher potato content (BR1), although all blends showed a notable reduction at 72 h. This trend is consistent with previous studies reporting that extended fermentation promotes microbial starch degradation, leading to decreased carbohydrate content (Anberbir et al. 2023). Similar studies on injera made from teff, sorghum, and faba bean have reported that shorter fermentation times help preserve higher carbohydrate levels, whereas extended fermentation leads to decreased carbohydrate content due to increased microbial activity. Furthermore, the blending of teff with other cereals or tubers generally lowers the carbohydrate content compared to pure teff injera, as supported by research on composite flour injera (Amtataw et al. 2025).

### 3.5 Microbial Analysis of Injera

The interaction effects of blending ratio and fermentation time on total bacterial count (TBC) as well as yeast and mold counts are presented in Table 6. The interaction between blending ratio (BR) and fermentation time (FT) significantly influenced the total bacterial count (TBC) across the storage period (*p* < 0.05). Generally, TBC increased substantially from Day 1 through Day 3 to Day 5, reflecting the expected proliferation of fermentative bacteria utilizing available carbohydrates in the injera batter (Berhanu et al. 2023).

**TABLE 6:** Interaction effects blending ratio and fermentation time on the microbial load of injera.

BR*t	Total bacteria count (logcfu/g)			Yeast and mold count (logcfu/g)		
	Day‐1 Day‐3 Day‐5			Day‐1 Day‐3 Day‐5		
BR1t1	3.37 ± 0.07^c^	4.73 ± 0.01^a^	7.44 ± 0.02^a^	3.21 ± 0.01^a^	3.85 ± 0.02^bc^	7.16 ± 0.12^a^
BR1t2	3.31 ± 0.33^c^	4.38 ± 0.07^b^	7.21 ± 0.09^ab^	2.82 ± 0.01^de^	3.23 ± 0.01^c^	7.15 ± 0.06^a^
BR1t3	3.65 ± 0.09^a^	4.28 ± 0.03^bc^	7.34 ± 0.10^ab^	2.97 ± 0.01^bc^	3.24 ± 0.09^c^	7.10 ± 0.03^a^
BR2t1	3.33 ± 0.08^ab^	4.40 ± 0.02^b^	7.44 ± 0.03^a^	2.86 ± 0.01^cd^	3.26 ± 0.015^bc^	6.93 ± 0.02^b^
RR2t2	3.17 ± 0.05^ab^	4.66 ± 0.10^a^	7.50 ± 0.10^a^	2.69 ± 0.03^ef^	3.24 ± 0.01^bc^	7.17 ± 0.01^a^
BR2t3	3.07 ± 0.10^b^	4.10 ± 0.13^c^	7.42 ± 0.01^a^	2.24 ± 0.01^g^	3.04 ± 0.02^d^	7.12 ± 0.11^a^
BR3t1	3.29 ± 0.05^ab^	4.40 ± 0.02^b^	7.34 ± 0.08^ab^	2.70 ± 0.01^ef^	3.24 ± 0.015^bc^	7.07 ± 0.03^ab^
RR3t2	3.36 ± 0.12^ab^	4.41 ± 0.02^b^	7.3 ± 0.11^ab^	2.70 ± 0.024^ef^	3.83 ± 0.01^a^	7.14 ± 0.01^a^
BR3t3	3.41 ± 0.02^ab^	4.32 ± 0.08^b^	7.36 ± 0.11^ab^	3.10 ± 0.02^a^	3.23 ± 0.02^d^	7.03 ± 0.01^ab^
BRCt1	3.27 ± 0.03^b^	4.37 ± 0.02^b^	7.42 ± 0.2^a^	2.86 ± 0.01^cd^	3.30 ± 0.02^bc^	7.18 ± 0.03^a^
BRCt2	3.39 ± 0.03^ab^	4.45 ± 0.01^b^	7.06 ± 0.24^b^	2.60 ± 0.02^f^	3.33 ± 0.04^b^	7.20 ± 0.07^a^
BRCt3	3.39 ± 0.03^ab^	4.38 ± 0.09^b^	7.46 ± 0.10^a^	3.07 ± 0.16^ab^	3.83 ± 0.01^a^	7.09 ± 0.02^ab^
CV	0.63	0.03	0.15	0.31	0.14	0.14
*p*‐Value	0.00	0.00	0.002	0.00	0.00	0.00

*Note:* Mean ± standard deviation; Means with different superscripts within a column differ significantly (*p* < 0.05). where: BR = blending ratio; BR1 = (70% Teff: 10% maize: 20% potato), BR2 = (70% Teff: 15% maize: 15% potato), BR3 = (70% Teff: 20% maize: 10% potato), BRC = (100% Teff:0% maize: 0% potato).

On Day 1, BR1T3 showed the highest initial TBC (3.65 log cfu/g), possibly due to the higher proportion of readily available starches from potato accelerating initial growth, especially when coupled with the longest fermentation start time (Fekadu Gemede 2015). Conversely, BR2T3 had the lowest count (3.07 log cfu/g), suggesting this specific combination might establish inhibitory conditions (faster initial acidification) or a less favorable nutrient profile for rapid initial bacterial growth compared to others (Leykun et al. 2023). By Day 3, the interaction effects were more pronounced. BR1T1 reached the highest TBC (4.73 and 4.66 log cfu/g). The readily available substrate in the high‐potato blend (BR1) likely allowed rapid proliferation even with shorter fermentation (T1), while the 72 h fermentation (T2) appeared optimal for the (BR2) to maximize bacterial load by this time point. Notably, BR2T3 exhibited the lowest TBC (4.10 log cfu/g), possibly because the extended fermentation led to earlier accumulation of inhibitory lactic acid or partial depletion of key nutrients, slowing the net growth rate observed at Day 3 compared to other treatments.

The 100% teff control (BRC) generally exhibited intermediate total bacterial counts (TBC) on Day 3. By Day 5, TBC increased across most treatments, reaching high levels (7.06–7.50 log cfu/g) table 6, likely approaching the carrying capacity of the substrate (Leykun et al. 2023), while many combinations showed statistically similar high counts (‘a’ or ‘ab’ superscripts), a significant interaction persisted, with BRCT2 (100% Teff, 72 h) showing a lower count (7.06 log cfu/g) than the peak observed in BR2T2 (7.50 log cfu/g). This suggests the BR2 blend fermented for 72 h supported a slightly higher final bacterial density than 100% teff under its optimal fermentation time (T2). Comparing these TBC results to the maximum permissible limit for ready‐to‐eat baked goods, which was 5.3 log cfu/g (Ummah 2019), it is observed that all injera samples on Day 1 and 3 were below this specific guideline limit. Therefore, based strictly on this TBC standard, the injera would be considered acceptable at Day 3. However, by Day 5, all samples substantially exceeded this limit.

Significant interaction effects between blending ratio and fermentation time were also observed for Yeast and Mold Count (YMC) at all time points (*p* < 0.05) Table 6. Yeast and mold counts (YMC) generally increased over the storage period, with a marked rise observed between Day 3 and Day 5, consistent with the role of yeasts in injera fermentation and subsequent proliferation (Amtataw et al. 2025). On Day 1, BR1T1 (high potato, 60 h) exhibited the highest YMC (3.21 log cfu/g), mirroring the total bacterial count trend and suggesting that readily available carbohydrates from potato may stimulate initial yeast activity (Steele et al. 2024). In contrast, BR2T3 (balanced, 84 h) showed the lowest count (2.24 log cfu/g), indicating that this specific combination was less favorable for early yeast proliferation.

By Day 3, the interaction pattern shifted. The highest YMC were observed in BR3T2 (70:20:10, 72 h) and BRCT3 (100% Teff, 84 h), both at 3.83 log cfu/g Table 6. This suggests that the higher maize content in BR3 combined with 72 h fermentation, or 100% teff with 84 h fermentation, provided optimal conditions for yeast growth by this stage, perhaps related to the specific carbohydrate profiles and moderate acidity levels148. BR2T3 (balanced, 84 h) consistently maintained the lowest YMC (3.04 log cfu/g) through Day 3, implying this blend/fermentation combination might exert a suppressive effect on yeast growth, possibly due to specific microbial competition or nutrient limitations unfavorable to yeasts under these conditions (Monti et al. 2024). At Day 5, YMC reached very high levels (6.93–7.20 log cfu/g). While most treatments resulted in similarly high counts (‘a’ or ‘ab’), BR2T1 (balanced, 60 h) had a significantly lower YMC (6.93 log cfu/g) compared to the peak in BRCT2 (100% Teff, 72 h) at 7.20 log cfu/g (Steele et al. 2024). This suggests that the shorter 60 h fermentation for the balanced blend may result in a slightly slower progression toward peak yeast density by Day 5, compared to other combinations such as 100% teff fermented for 72 h, which exhibited the highest final yeast count. Comparing the YMC results to the standard for RTE baked goods (< 4.0 logfu/g) (Saddozai et al. 2009), all injera samples on Day 1 and Day 3 (with the highest Day 3 value being 3.85 log cfu/g) remained within this guideline limit1. This indicates that yeast and mold counts remained within acceptable limits up to Day 3 according to the specified standard. However, by Day 5, all samples exceeded this threshold (lowest value: 6.93 log cfu/g), suggesting significant yeast proliferation and potential spoilage (Amtataw et al. 2025).

### 3.6 Mineral Content Analysis of Injera

The effects of blending ratio and fermentation time on the mineral content of injera are presented in Table 7. Calcium is an essential nutrient that plays a vital role in blood clotting and providing structural rigidity to the skeleton through its phosphate salts (Theobald 2015). The interaction of blending ratio and fermentation time showed a significant (*p* < 0.05) effect on the calcium content of the samples. The values varied across different blending ratios and fermentation periods, with the highest calcium content observed in BRC and the lowest in BR3 at 60 h. This result is consistent with previous studies, which found that the addition of other grains, such as maize and potato, significantly reduced the calcium content of injera due to the naturally higher calcium levels in teff compared to these grains (Ashenafi 2016).

**TABLE 7:** Effect of blending ratio and fermentation time on the calcium, iron and zinc content of injera.

	BR	BRC	BR1	BR2	BR3	CV	*p*
Calcium Iron Zinc	60 h	45 ± 2.0^c^	32 ± 1.5ᵉ	24 ± 1.2ᵍ	15 ± 1.0ʰ	4.5	< 0.001
	72 h	47 ± 2.2ᵇ	34 ± 1.6ᵈ	26 ± 1.3ᶠ	18 ± 1.1ᵍ	4.5	< 0.001
	84 h	57 ± 2.5ᵃ	34 ± 1.6ᵈ	28 ± 1.4ᶠ	23 ± 1.2ᵍ	4.4	< 0.001
	60 h	70 ± 2.5ᶜ	66 ± 2.4ᵇ	60 ± 2.2ᶠ	45 ± 2.0ʰ	3.5	< 0.001
	72 h	75 ± 2.8ᵃᵇ	69 ± 2.6ᶜ	62 ± 2.3ᵉ	50 ± 2.2ʰ	3.6	< 0.001
	84 h	76 ± 2.9ᵃ	72 ± 2.7ᵇ	65 ± 2.4ᶠ	53 ± 2.3ʰ	3.7	< 0.001
	60 h	21 ± 1.2ᶜᵈ	26 ± 1.3ᵇᶜ	16 ± 1.0ᶠ	14 ± 0.9ʰ	5.0	< 0.001
	72 h	23 ± 1.3ᵇ	27 ± 1.4ᵃᵇ	18 ± 1.1ᵉ	15 ± 1.0ᵍʰ	5.0	< 0.001
	84 h	24 ± 1.4ᵃ	27 ± 1.5ᵃ	19 ± 1.2ᵈ	16 ± 1.1ᶠᵍ	5.1	< 0.001

*Note:* Mean ± standard deviation; Means with different superscripts within a column differ significantly (*p* < 0.05). where: BR = blending ratio; BR1 = (70% Teff: 10% maize: 20% potato), BR2 = (70% Teff: 15% maize: 15% potato), BR3 = (70% Teff: 20% maize: 10% potato), BRC = (100% Teff:0% maize: 0% potato).

Microbial activity, particularly lactic acid bacteria (LAB) and yeasts, produces organic acids such as lactic and acetic acids. These acids help break down phytic acid, an anti‐nutrient that binds minerals and inhibits their absorption. As phytic acid degrades, calcium becomes more bioavailable, leading to an increase in measurable calcium content (Baye 2014). Additionally, phytase enzymes naturally present in teff or produced by microbes enhance calcium release from bound forms, further contributing to the observed increase Abebaw Tsegaye (2020); Bikila (2020). As fermentation time increased from 60 to 84 h, the calcium (Ca) concentration also increased. This is likely due to the breakdown of anti‐nutritional factors, such as phytates, and enhanced mineral solubility, which improve calcium availability in the dough (Dessie 2023), contrary to the findings (Baye 2014), who reported a decrease in calcium content with excessive fermentation.

In addition to that, the statistical analysis indicated significant differences among the samples, with BRC showing the highest calcium levels, followed by BR1, BR2, and BR3. The observed decline in calcium concentration as maize and potato proportions increased suggests that these ingredients decrease the calcium content compared to teff injera. These findings highlight the importance of maintaining a higher teff proportion in composite blends to preserve calcium levels during fermentation. (Asrat 2021).

The nutritional contribution of iron, crucial for systemic functions like oxygen transport and enzymatic processes, was significantly influenced (*p* < 0.05) by both the ingredient blend and the duration of fermentation (60, 72, 84 h) in the samples studied. A primary observation is the pronounced impact of the blend composition. The blends analyzed were BRC, BR1, BR2, and BR3. Iron concentrations systematically decrease across these blends, moving from the highest in BRC at 84 h (75.70 mg/100 g) to the lowest in BR3 for 60 h (45.97 mg/100 g) Table 7. This trend strongly supports the well‐documented high iron content inherent to teff being progressively reduced by the increasing inclusion of maize and potato, ingredients known to possess lower iron levels (Mihrete and Bultosa 2017).

In addition to this compositional effect, the dynamic influence of fermentation time was observed. Within each blend, fermenting for 72 h (t2) consistently resulted in the highest, or near‐highest, iron concentrations compared to 60 h (t1) or 84 h (t3). This peak around 84 h likely reflects the positive impact of microbial metabolism. Lactic acid bacteria and yeasts generate organic acids, lowering pH and enhancing the breakdown of phytate–mineral complexes, thereby increasing iron solubility (Endalew et al. 2024). The action of phytase enzymes, both endogenous and microbial, further liberates iron, while the reducing environment potentially favors the more soluble ferrous (Fe2+) state (Satheesh and Fanta 2018).

This result shows how mixing different amounts of teff, maize, and potato, and fermenting for different times, affects the zinc (Zn) level in Injera. As fermentation time increases from 60 to 84 h, zinc levels in injera also increase across all blending ratios, as clearly shown in Table 7. The highest zinc levels were observed in the BR1 blend at 84 h of fermentation time (27.07 mg/100 g), while the lowest levels were consistently seen in the BR3 blend at 60 h fermentation time (14.07 mg/100 g) Table 7, which likely had the most maize. This rise in zinc content with longer fermentation is likely due to the ongoing breakdown of phytic acid and other anti‐nutritional factors by fermenting microorganisms, which releases more zinc and enhances its bioavailability (Nyachoti et al. 2021).

Extended fermentation encourages the growth of beneficial lactic acid bacteria that produce organic acids, further helping to solubilize and release zinc from the food matrix, making it more accessible for absorption (Leeuwendaal et al. 2022). This pattern is consistent with other research findings that demonstrate increased mineral availability with prolonged fermentation in cereal‐based foods (Serpen et al. 2008). In short, the mix of ingredients was the main factor deciding the zinc level, with potato adding the most zinc and maize adding the least compared to teff. The fermentation process helped increase available zinc for up to about 84 h, with zinc levels rising steadily throughout this period. This suggests that both the choice of ingredients and the length of fermentation are important for maximizing the zinc content in Injera. In short, the mix of ingredients was the main factor deciding the zinc level, with potato adding the most zinc and maize adding the least compared to teff. The fermentation process helped increase available zinc in all fermentation time Table 7.

### 3.7 Sensory Evaluation of Injera

**FIGURE 1:**
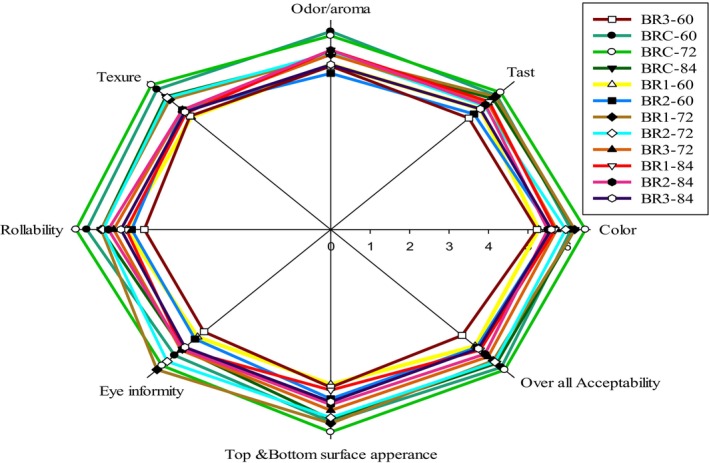
Sensory acceptability of injera from composite flours.

A radar chart in Figure 1 illustrates the results of the sensory evaluation of injera made from multiple teff, maize, and potato blending ratios over different fermentation periods. The analysis compares three experimental blends (BR1, BR2, and BR3) against a control sample (BRC) across multiple sensory attributes. The control sample (BRC) consistently exhibited the highest color ratings across all fermentation times, with BRC at 72 and 84 h achieving the top scores. Among the experimental blends, BR1 exhibits better color acceptance than BR2 and BR3, particularly at longer fermentation times (72 and 84 h). The presence of maize appears to negatively impact color perception, as BR3 (with highest maize content) consistently shows lower color ratings (Anberbir et al. 2023). This aligns with research finding that teff‐based injera generally scores higher in color acceptance, though studies have shown that blends with 20% potato and 80% red teff can achieve good color ratings as consumers appreciate the lighter appearance contributed by potato starch (Yassin and Getu 2019).

Taste evaluation shows a clear preference for the control (BRC) samples fermented for 72 and 84 h: Figure 1. Among the experimental blends, BR1 (with highest potato content) demonstrates superior taste ratings, especially at 84 h fermentation (Yassin and Getu 2019). The longer fermentation period generally enhances taste characteristics across all samples, likely due to the development of desirable sour flavors from increased lactic acid production (Anberbir et al. 2023). Recent research confirms that fermentation time significantly impacts taste, with 84 h fermentation usually producing more preferred taste profiles due to optimal acid development.

The control samples exhibit the strongest odor/aroma ratings, particularly at 72 and 84 h fermentation. Among the blends, BR1 shows better aroma ratings than other experimental samples. Fermentation time positively influences aroma development, with 84 h generally yielding more acceptable results than 60 h across all samples. This aligns with studies showing that longer fermentation allows for better development of characteristic injera aroma compounds through microbial activity.

Texture evaluation shows a notable preference for the control samples, with BRC‐72 h and BRC‐84 h achieving the highest ratings. Among experimental blends, BR1 (highest potato content) at 84 h fermentation demonstrates the best texture, followed by BR2. The high texture scores for potato‐containing blends align with research showing that proper incorporation of potato can improve injera softness and pliability. Studies have found that the starch contribution from potato can enhance textural properties, particularly when fermented adequately (Yassin and Getu 2019).

Roll‐ability is following a similar pattern to texture, with control samples showing superior performance. The experimental blends show lower rollability than the control, with BR3 (highest maize content) demonstrating the poorest performance. Research indicates that higher maize content tends to reduce rollability due to the gluten‐free nature of maize and its impact on the structural integrity of injera. The potato content in BR1 appears to partially mitigate this effect, as it shows better rollability than BR2 and BR (Yassin and Getu 2019).

The control samples show the best eye uniformity (characteristic holes on injera surface), particularly at 72 and 84 h fermentation: Figure 1. Among the blends, BR1 at 84 h performs relatively well. Research confirms that proper eye formation is critical for injera quality, with longer fermentation times typically yielding better results due to adequate gas production during fermentation (Anberbir et al. 2023). Surface appearance ratings are highest for the control samples at 72 and 84 h fermentation. Among the experimental blends, BR1 consistently outperforms BR2 and BR3 across all fermentation times. This aligns with research showing that surface characteristics are heavily influenced by both blend composition and fermentation duration. Studies indicate that proper fermentation allows for optimal CO2 production and structural development, which contributes to desirable surface appearance (Ashenafi 2006).

The control sample at 72 and 84 h fermentation shows the highest overall acceptability, confirming the traditional preference for properly fermented teff injera. Among the experimental blends, BR1 at 84 h fermentation demonstrates the best overall acceptability. This finding is consistent with research showing that while 100% teff: 0% maize: 0% potato injera is generally preferred, properly formulated blends with potato can achieve good consumer acceptance. Yassin and Getu (2019), studies have found that blends containing 20% potato with teff can achieve high consumer acceptance, which aligns with the relatively good performance of BR1 in this evaluation.

Overall, the sensory evaluation demonstrates that while BRC injera remains superior across most attributes, properly formulated blends with appropriate fermentation times can achieve acceptable quality. The blend with the highest potato content (BR1) consistently outperforms other experimental formulations, and longer fermentation times (72 and 84 h) yield better sensory attributes across all samples.

## 4 Conclusions

Teff is high in fiber and protein, maize is rich in fat and carbohydrates, and potato provides high moisture and protein but low fat and fiber. Blending ratios and fermentation times significantly influenced injera's proximate composition, titratable acidity, and microbial counts. The BR1 fermented for 72–84 h showed the best balance of nutrition, texture (softness and rollability), and consumer acceptability, closely matching the quality of 100% teff injera. Partial substitution of teff with maize and potato, combined with controlled fermentation, can produce safe, high‐quality injera that supports food security, dietary diversity, high sensory acceptability and cost reduction, with microbial safety maintained up to 3 days at room temperature. Overall, the findings suggest that partial substitution of teff with maize and potato, along with fermentation time (72 h), can yield injera that meets good quality, nutritional, and sensory expectations.

## Author Contributions

**Aynadis Molla Asemu:** writing – review and editing. **Yengus Lake Cherinet:** conceptualization, methodolgy. **Birhanu Ayitegeb Ambaw:** investigation, Formal analysis. **Wendu Hilemichael Hilemeskel:** software. **Mesfin Wogayehu Tenagasahw:** writing – review and editing, supervising. **Zenamarkos Bantie Sendekie:** visualization. **Behailu Bisenebit Mossie:** writing – original draft, writing – review and editing.

## Funding

The authors have nothing to report.

## Ethics Statement

The study complies with all country regulations for sensory evaluation and was conducted according to established ethical guidelines. Ethical clearance was obtained from Bahir Dar Institute of Technology Institutional Research Ethics Review Board, with approval number BiT‐IRERB/067/2026. The panelists provided informed written consent.

## Conflicts of Interest

The authors declare no conflicts of interest.

## Data Availability

The data that support the findings of this study are available from the corresponding author upon reasonable request.
